# A rare combination of an axillary artery and brachial plexus injury due to a proximal humeral fracture

**DOI:** 10.4103/0973-6042.59975

**Published:** 2009

**Authors:** Robin M Seagger, Jeffery Kitson

**Affiliations:** The Orthopaedic Department, The Royal Devon and Exeter Hospital, Barrack Road, Exeter, EX25DW, United Kingdom

Sir,

We present a case of an 88-year-old woman who presented to the Emergency Department with an injury to her dominant right shoulder. She fell forwards catching her upper arm between two railings. The paramedics at the scene reported a cold pulseless hand that was insensate and lacking all power of elbow and hand movement. At admission to the Emergency Department, she had regained normal sensation in the limb; however, she was left with a dense paralysis of her elbow, wrist and hand, suggesting radial, median and ulnar nerve involvement. There were no palpable pulses, and Doppler failed to identify a brachial, radial or ulnar pulse. Despite this, the limb was warm and not critically ischemic, with a capillary refill of less than 2 seconds.

**Figure 1:**
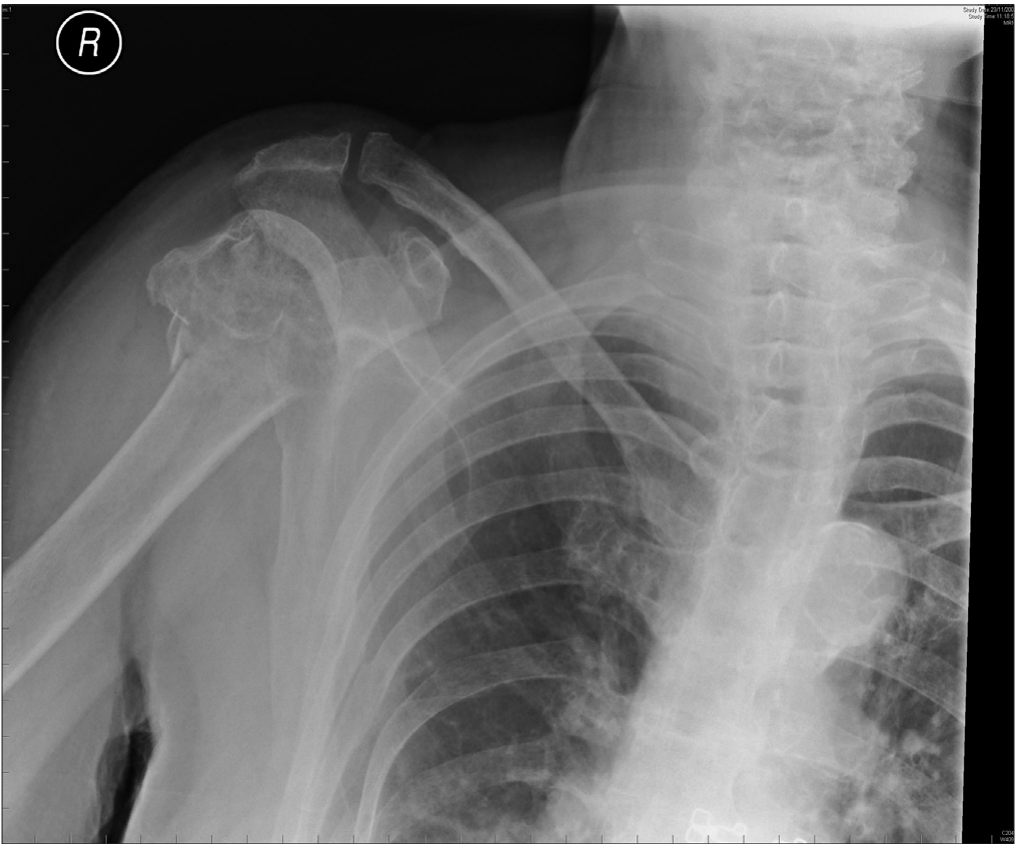
Plain AP radiograph of right shoulder. A ‘Neer’ 2-part fracture is seen

**Figure 2:**
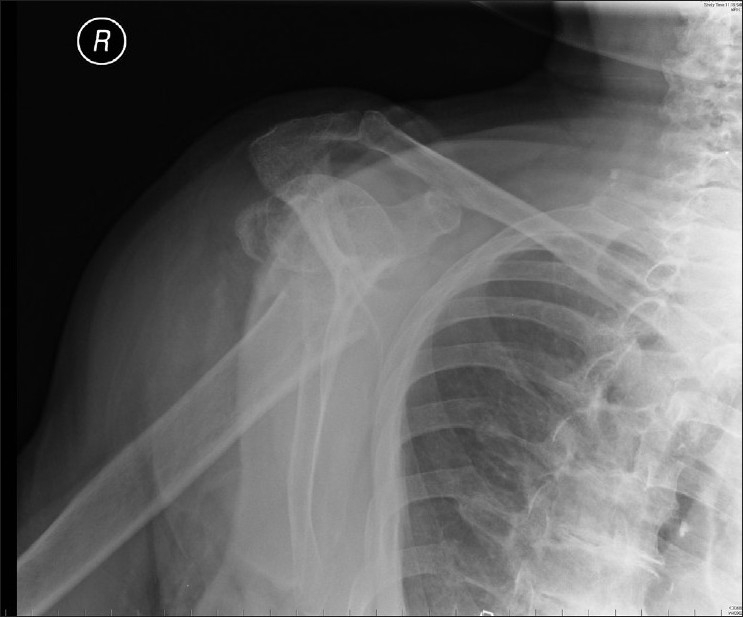
Plain lateral scapula radiograph of right shoulder. A ‘Neer’ 2-part fracture is seen

Plain radiographs showed a Neer 2-part proximal humeral fracture with significant comminution, displacement and angulation [Figures 1 and 2]. It was assumed the neurological abnormalities were due to a brachial plexus injury, given the level of the fracture.

**Figure 3:**
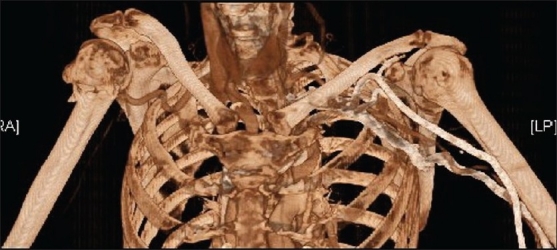
Venous contrast computerized tomography scan. Contrast can clearly be seen passing proximally in the left arm veins, passing through the heart and filling the right subclavian artery. There is no contrast beyond the level of injury

Emergency imaging of the presumed vascular injury was organized. Standard angiography was unavailable, so a 'venous contrast' computerized tomography (CT) angiogram was requested [Figure 3]. The scan confirmed the nature of the fracture, and showed the axillary artery was 'tented' within the fracture and occluded at this level. There was no obvious compressive hematoma at the level of the injury to account for the neurological findings.

Discussion with the vascular surgeons initially suggested that the vascular injury could be treated conservatively, relying on collateral circulation, as the limb was warm and the brachial plexus injury was not due to pressure effect from a large hematoma.

A review of the literature, as presented later, and further discussions with the orthopedic and vascular surgeons resulted in a joint procedure to explore the brachial plexus, reduce and plate the fracture and reconstruct the artery.

At surgery a standard delto-pectoral approach was utilized. A coracoid osteotomy was undertaken to expose the brachial plexus. The plexus and artery were dissected from above the zone of injury to the main terminal branches. The brachial plexus and branches were grossly intact, with no external injury. The axillary artery, in contrast, was 'caught' around a spike of bone from the distal shaft fragment and displayed a clear area of contusion, and intraoperative Doppler at this level demonstrated no flow through this 'zone of injury.' The artery was released and the fracture was reduced to an acceptable position and stabilized with an Arbeitsgemeinschaft Osteosynthesefragen (AO) PHILOS (proximal humeral interlocking system) plate. The vascular surgeons then performed a longitudinal arteriotomy, thrombectomy and excised a small intimal tear. The artery was then repaired using a small vein patch.

Immediately postoperatively, the patient had a strong and easily palpable radial pulse in the affected upper limb. Sensation was present but slightly reduced globally. A dense paralysis remained below the elbow.

During the first postoperative week, she regained normal sensation in the entire upper limb. The median nerve motor function began to recover; and after 5 days, she had Medical Research Council (MRC) grade II in the median nerve myotome of her right hand. At 6 days she had recovered to MRC grade IV elbow flexion, grade IV wrist flexion and grade III finger flexion with improving excursion. Elbow and wrist extensions were still absent. Radial pulse remained strong.

**Figure 4:**
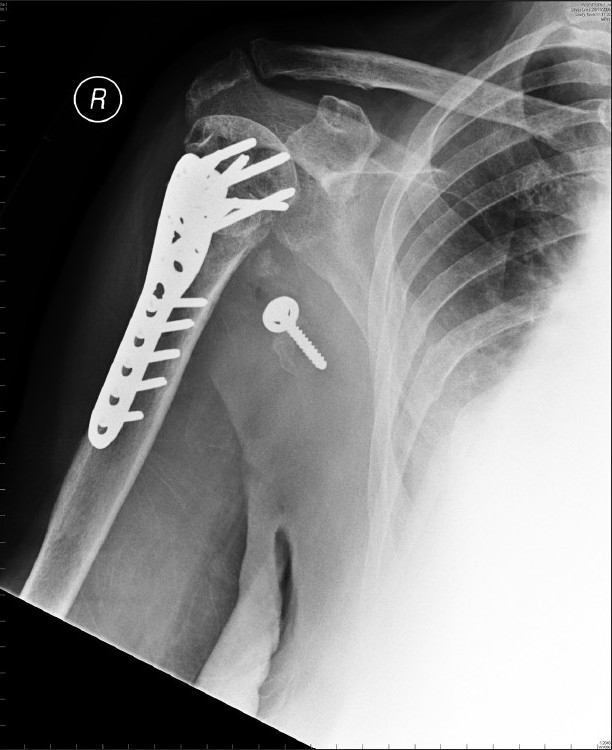
Antero-posterior radiograph at 1 year. Proximal humerus has healed. Coracoid has displaced

One year post-injury, she continued to show signs of neurological recovery. Sensation in the limb remained normal. She demonstrated grade IV power of elbow flexion and some recovery of extension, grade II. The fracture had healed well. The coracoid had displaced [Figure 4].

Proximal humeral fractures remain a common presentation in orthopedic practice, accounting for up to 5% of all fractures.[1] The majority (84%) of these patients are over 50 years old,[1] and only 6% require any form of fixation or replacement.[1] There is, however, a rare subgroup of patients that have an associated vascular injury to the axillary artery, with an incidence of 5%.[2] Brachial plexus injury is a slightly more common finding, viz., in 6% of patients;[2] however, the prevalence of a brachial plexus injury is much higher — approximately 50% — in the presence of a concomitant arterial injury.[1]

The vast majority (89%) of traumatic injuries to the axillary artery occur at the level of the third part of the vessel.[3] This is thought to be due to the fact that this part of the artery is tethered by the anterior and posterior circumflex vessels.[3] Clearly, however, trauma from a sharp bony fragment can occur at any level.

There are numerous anatomises around the proximal humerus — between the transverse cervical, suprascapular and subscapular vessels. These can result in the appearance of a well-perfused limb despite the presence of a significant vascular injury. Palpable distal pulses may be present in as many as 27% of serious arterial injuries,[4] which may result in misdiagnosis or under-treatment of a potentially limb-threatening injury.

Amputation rate after acute vascular repair is 21%.[2] If there is delay in presentation or diagnosis, this rate could be as high as 50%.[5] In the presence of a displaced proximal humeral fracture, a high index of suspicion of both neurological and vascular injuries should be maintained. Vascular injury may still be present despite palpable distal pulses and a warm hand.[3] If a vascular injury is suspected, emergency imaging may help diagnose a developing hematoma, vessel rupture or occlusion. In an ischemic limb, conservative management is clearly contraindicated. In a limb with a proven vascular injury but adequate distal perfusion, fracture stabilization and vascular repair are likely to improve long-term outcome.[3] The conservative management of axillary artery injuries in the presence of a proximal humeral fracture is not recommended.[1]
